# Influence of disabilities on state‐level disparity in dental care utilization and tooth loss: Findings from the 2020 Behavioral Risk Factor Surveillance System

**DOI:** 10.1111/scd.12944

**Published:** 2023-11-28

**Authors:** Reem Binmogren, Aljohara Alshathri, Habah Alhamdan, Rand Alnuwaiser, Muath Aldosari

**Affiliations:** ^1^ College of Dentistry King Saud University Riyadh Saudi Arabia; ^2^ Periodontics and Community Dentistry King Saud University College of Dentistry Riyadh Saudi Arabia; ^3^ Oral Health Policy and Epidemiology Harvard School of Dental Medicine Boston Massachusetts USA; ^4^ Health Policy and Health Services Research Boston University Boston Massachusetts USA

**Keywords:** access to dental care, adults with disability, dental care utilization, dental outcomes, oral health disparities, tooth loss

## Abstract

**Introduction:**

The aim of this study is to describe the prevalence of disparity in access to dental care and tooth loss between US adults with and without disabilities at the state level.

**Methods:**

This secondary analysis included data from the 2020 Behavioral Risk Factor Surveillance System (BRFSS), a cross‐sectional state‐run annual telephone survey of noninstitutionalized US adults aged 18 years or older. The primary predictor, having any disability, was defined as reported difficulty in hearing, vision, cognition, mobility, self‐care, or independent living. The two dental outcomes used were reported time since last dental visit and missing teeth status. We conducted descriptive analysis and multinomial regression models on weighted data to report the prevalence and state‐level disparity in dental outcomes between individuals with and without disability.

**Results:**

Nationally, one in four adults reported a disability, with a disproportionately higher prevalence among females, Native Americans and lower education and income groups. The highest utilization of dental services within a year among individuals with disabilities was found in the Northwestern and Midwestern states. Western states had the highest proportion of individuals with disability to have had a recent dental visit and a complete dentition, while the Southern states had the lowest proportions. After adjusting for sociodemographic factors, 59.1% of individuals with disabilities had dental visits within a year, which is 8.0% points less than those without disabilities. Similarly, only 50.8% of individuals with a disability had complete tooth retention, which is 10.1% points less than individuals without disability. Regardless of the type of disability, adults with a disability were less likely to have visited the dentist in the past year or to have retained all of their teeth. Those with self‐care disabilities had the lowest rates of both.

**Conclusion:**

There were clear disparities in the utilization of dental care and dentate status among adults with disabilities at the state level. The findings from this study highlighted the necessity of finding local solutions to address the gap in dental care utilization and tooth loss between those with and without disability among the US population.

## 1 INTRODUCTION

Disability is a complex concept that can be defined in many ways. The World Health Organization (WHO) defines disability as “A restriction or lack of ability (resulting from an impairment) to perform an activity in the manner or within the range considered normal for a human being.” In a report published in 2018, 25.7% of American adults stated that they had a disability.^1^ According to 2019 BRFSS data, of all the disability types, mobility was the most common, affecting 13.3% of people. Cognition was the second most common, affecting 12.1% of people. Hearing, vision, and self‐care were less common, affecting 6.1%, 5.2%, and 3.8% of people, respectively.^1^

While daily life activities could be challenging, people with disabilities often face additional obstacles that can make it more difficult to overcome them. These obstacles can be related to physical access, social attitudes, or economic resources.^2^ In health care, people with disabilities face more challenges accessing health services than people without disabilities.^3^ Cost often presents a significant challenge to accessing healthcare; however, this issue is markedly magnified among individuals with disabilities, as their healthcare needs tend to be higher, which consequently escalates their overall healthcare expenditure.^3^ Similarly, transportation and structural barriers have been a particularly complex challenge for individuals with disabilities, with one study demonstrating that women with multiple disabilities are more likely to encounter issues with transportation, parking, and accessibility challenges.^4^ Moreover, these women often report a lack of physician recommendations for necessary screenings.^4^

The National Institute of Dental and Craniofacial Research (NIDRC) reports that people with disabilities are at higher risk for oral diseases, including but not limited to: tooth decay, periodontitis, malocclusion, delayed tooth eruption, and trauma.^5^ A systematic review by Anders and Davis in 2010 concluded that people with intellectual and developmental disabilities have higher prevalence and severity of tooth decay and periodontal disease compared to those without disabilities due to the difficulties they face performing routine effective brushing and flossing.^6^ Regardless of oral health habits, other problems such as oral malformations and delayed tooth eruption may accompany some disabilities. Additionally, individuals with disabilities are more likely to take medications that can have oral implications. As a result, timely access to oral health care for individuals with disabilities, who present with less‐than‐ideal dental hygiene and an increased rate of oral disorders, is limited due to structural and systemic barriers.^7^ International studies, including those from Canada and Norway, have also demonstrated a higher prevalence of tooth loss and lower utilization of dental care services among people with disabilities.^8^, ^9^ Therefore, a main goal of the Healthy People 2030 initiative, recognized by the Office of Disease Prevention and Health Promotion, is to ensure that more people with disabilities can access needed health care services.^10^

The oral health status of people with disabilities has been extensively studied, but few studies have explored the gap in dental care utilization and tooth loss using national data. Additionally, given the varying degree of adult dental benefits across states, it is crucial to explore the state‐level disparities in oral health among people with disabilities. Therefore, the aim of this study was to explore the disparities in access to dental services and in tooth loss between US adults with and without disabilities in different states using data from the 2020 Behavioral Risk Factor Surveillance System (BRFSS). The results of this study will help to inform public advocacy efforts to close the gap in oral health associated with disabilities.

## 2 MATERIALS AND METHODS

### 2.1 Study design

BRFSS is an annual, state‐based, cross‐sectional, and thorough telephone survey conducted in partnership with the Centers for Disease Control and Prevention (CDC). The survey consists of core questions, optional modules, and state‐added questions to collect representative information regarding health conditions, behaviors, and the utilization of preventive measures for non‐institutionalized US civilians aged 18 years or older. The BRFSS data was collected with CDC ethics review board (ERB) approval and participants provided verbal consent. The ERB approved the initial data collection methods and ensured the data was de‐identified before being made publicly accessible. The analysis included 369 486 adult participants from all 50 states and the District of Columbia who reported their disability status and completed the oral modules questionnaire in the BRFSS survey.

### 2.2 Definition of disabilities and confounding factors

To define the main independent variable, we followed the guidance of the Division of Human Development and Disability in assessing disability status using BRFSS data.^11^ Participants were asked the following six questions to encompass six domains of disability: hearing, “Do you experience deafness or have significant difficulty hearing?”; vision, “Do you experience blindness or have significant difficulty seeing, even with corrective lenses?”; cognition, “Due to a physical, mental, or emotional condition, do you encounter significant difficulty concentrating, remembering, or making decisions?”; mobility, “Do you face significant difficulty walking or climbing stairs?”; self‐care, “Do you encounter difficulty in dressing or bathing?”; and independent living, “Due to a physical, mental, or emotional condition, do you find difficulty in running errands alone, such as visiting a doctor's office or shopping?” If participants answer “yes” to at least one of these questions, they are considered to have a disability. Additionally, we calculated the number of disabilities that each participant reported.

The analysis took into account the following confounding demographic factors: age (18–24, 25–34, 35–44, 45–54, 55–64, and 65+), sex (male, female), race/ethnicity (non‐Hispanic White, non‐Hispanic Black, non‐Hispanic Asian, Hispanic, other races—including Native Americans), income level (<$15 000, $15–24 999, $25–34 999, and $35 000+), and education level (less than high school, completed high school/GED, and education beyond high school).

### 2.3 Defining access to dental services and tooth loss

Access to dental care was assessed by inquiring about the participants’ most recent dental visit for any reason, including visits to orthodontists, oral surgeons, and all other dental specialists, as well as dental hygienists. Based on their response, we categorized the answers as “within the past year”, “1–5 years”, or ’’5+ years or never“. For the second outcome, participants were asked about tooth decay or gum disease‐related tooth removal to determine their number of missing teeth, which includes wisdom teeth if they were removed for these reasons. We collapsed the responses into ”none“, ”some“, and ”all’’.

### 2.4 Statistical analysis

Initially, we conducted a descriptive analysis to investigate the disability distribution across sociodemographic factors. We used Chi‐square to test the association between sociodemographic factors and the presence of disability. To account for the complex survey design and nonresponse bias, we used a weighting procedure, opting to use the suggested survey weights supplied in the dataset to represent the US adult population.

We then estimated the crude national and state‐level prevalence of disability, dental visits within a year, and complete teeth retention. After that, we used multiple multinomial logistic regression models to calculate state‐level adjusted proportions for visiting the dentist within the past year and the full teeth retention rate for people with disabilities and people without disabilities, adjusting for sociodemographic confounders. Lastly, we reported the national prevalence of each disability and the proportion who had access to dental care and complete teeth retention, stratified by disability status. All statistical analyses were performed using Stata/MP V.18.0 (StataCorp).

## 3 RESULTS

**FIGURE 1:**
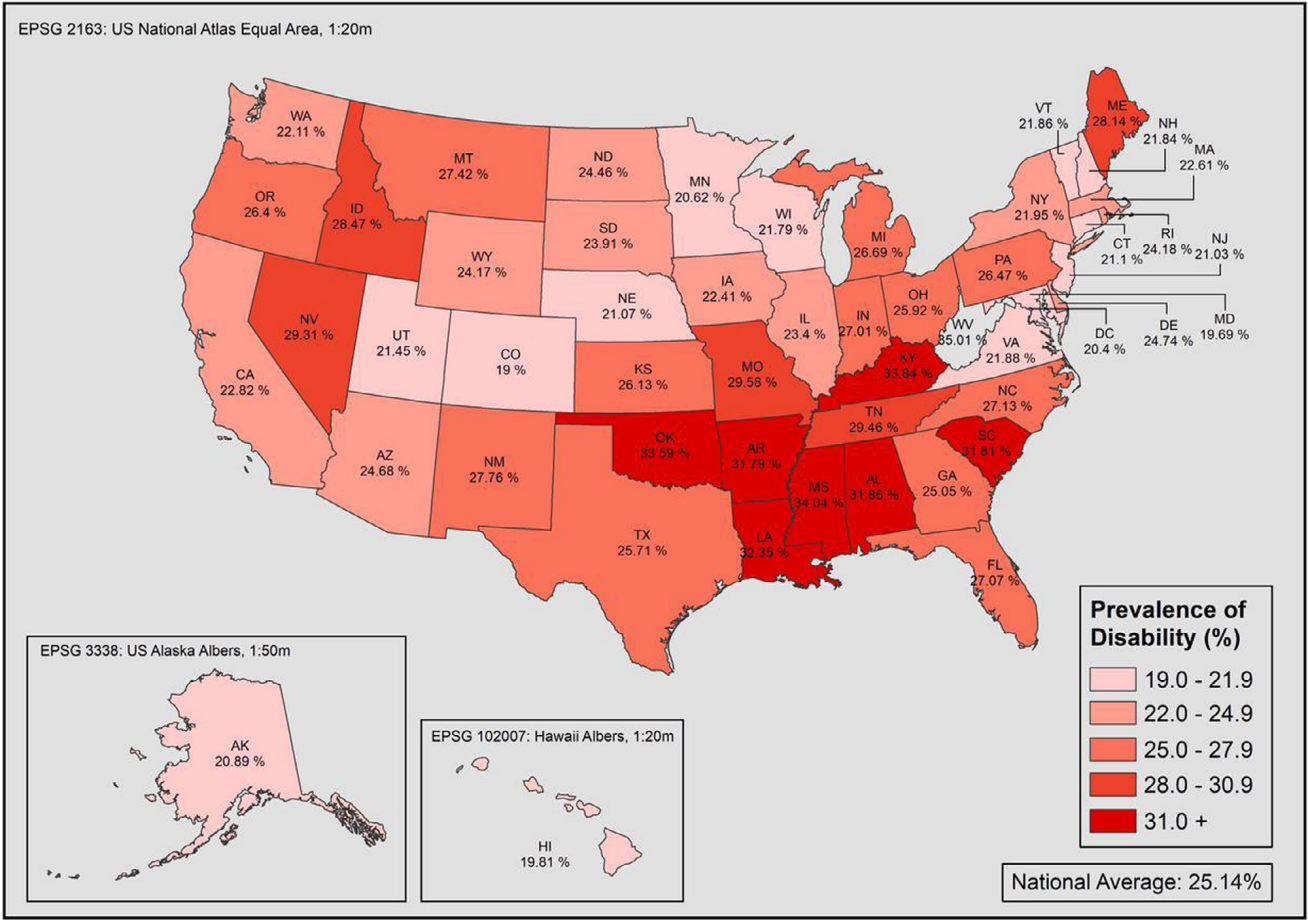
Prevalence of adults with disabilities by state in the United States in 2020.

Nationally, one out of four adults, which is about 60.3 million individuals, reported having some form of disability (Table 1). The highest proportion of adults with disability was among females (26.8%), the elderly aged 65 or older (40.0%), and one out of three individuals who identified as “Other” racial minorities, including Native Americans (34.0%). Disability was least reported among Asians (11.6%). Furthermore, the proportion of individuals reporting disabilities increased as education and income level decreased. All demographics were statistically associated with disability (*p*‐value <.05). At the state level, the highest prevalence of any disability reported was in the Southern states, followed by Nevada, Maine, and Idaho (Figure 1).

**TABLE 1:** Prevalence of disability by age, sex, race/ethnicity, education, and income among adults who reported their disability status and completed the oral modules questionnaire in the Behavioral Risk Factor Surveillance System (BRFSS), 2020.

		With disability		Without disability		
	Overall no (%)^a^	Weighted U.S. population N (in thousands)	Weighted percentage (%)	Weighted U.S. population N (in thousands)	Weighted percentage (%)	*P*‐value
Overall	**369 486 (100)**	**60 304**	**25.1**	**179 528**	**74.9**	
Age						<0.001
18–24	23 640 (6.4)	5658	19.5	23 438	80.5	
25–34	40 998 (11.1)	7315	17.4	34 806	82.6	
35–44	47 972 (13.0)	6769	17.2	32 646	82.8	
45–54	56 815 (15.4)	8249	21.7	29 753	78.3	
55–64	72 399 (19.6)	11 675	29.4	27 993	70.6	
65+	127 662 (34.6)	20 639	40.0	30 892	60.0	
Sex						<0.001
Male	169 720 (46.0)	27 484	23.5	89 649	76.5	
Female	199 766 (54.1)	32 820	26.8	89 879	73.3	
Race/ethnicity						<0.001
Non‐Hispanic White	285 631 (77.3)	30 039	25.2	113 214	74.9	
Non‐Hispanic Black	27 798 (7.5)	7577	27.1	20 438	73.0	
Non‐Hispanic Asian	8722 (2.4)	1525	11.6	11 660	88.4	
Hispanic	28 871 (7.8)	10 646	26.6	29 336	73.4	
Other race, including Native Americans	18 464 (5.0)	2518	34.0	4881	66.0	
Education						<0.001
Less than high school	22 854 (6.2)	12 369	43.7	15 955	56.3	
High school	96 657 (26.3)	18 940	29.0	46 439	71.0	
More than high school	248 716 (67.5)	28 818	19.6	116 338	80.2	
Income						<0.001
<$15 000	23 517 (7.7)	8749	49.4	8967	50.6	
$15–24 999	44 563 (14.6)	11 445	39.4	17 607	60.6	
$25–34 999	29 276 (9.6)	5873	32.3	12 316	67.7	
$35 000+	207 745 (68.1)	22 032	16.6	110 449	83.4	

^a^ The sample counts were unweighted, while percentages are weighted to account for complex survey design. The weighted population counts are rounded to the nearest 1000.

### 3.1 Dental care utilization

**FIGURE 2:**
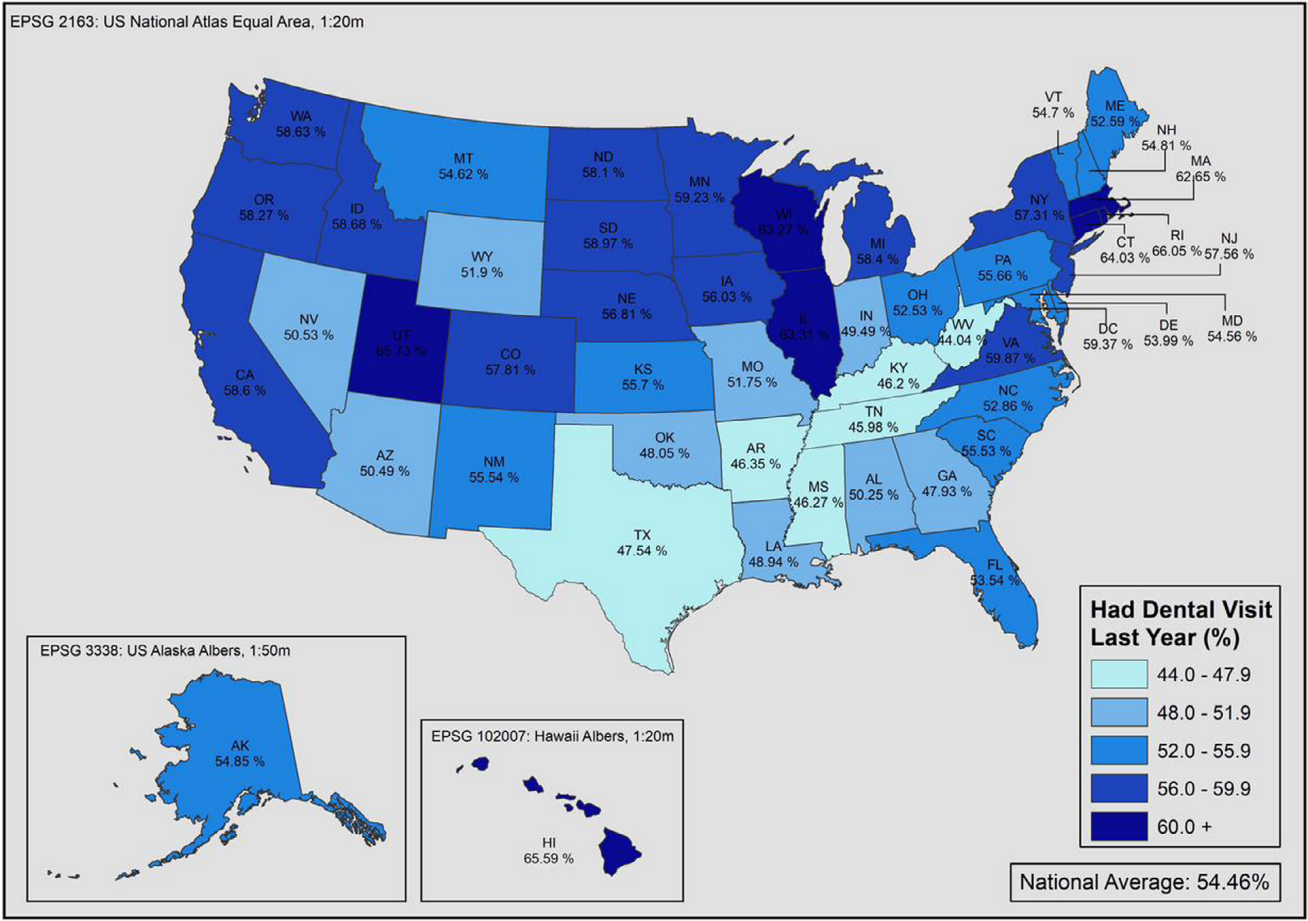
Proportion of adults with disabilities who had a dental visit within a year in the United States in 2020.

**FIGURE 3:**
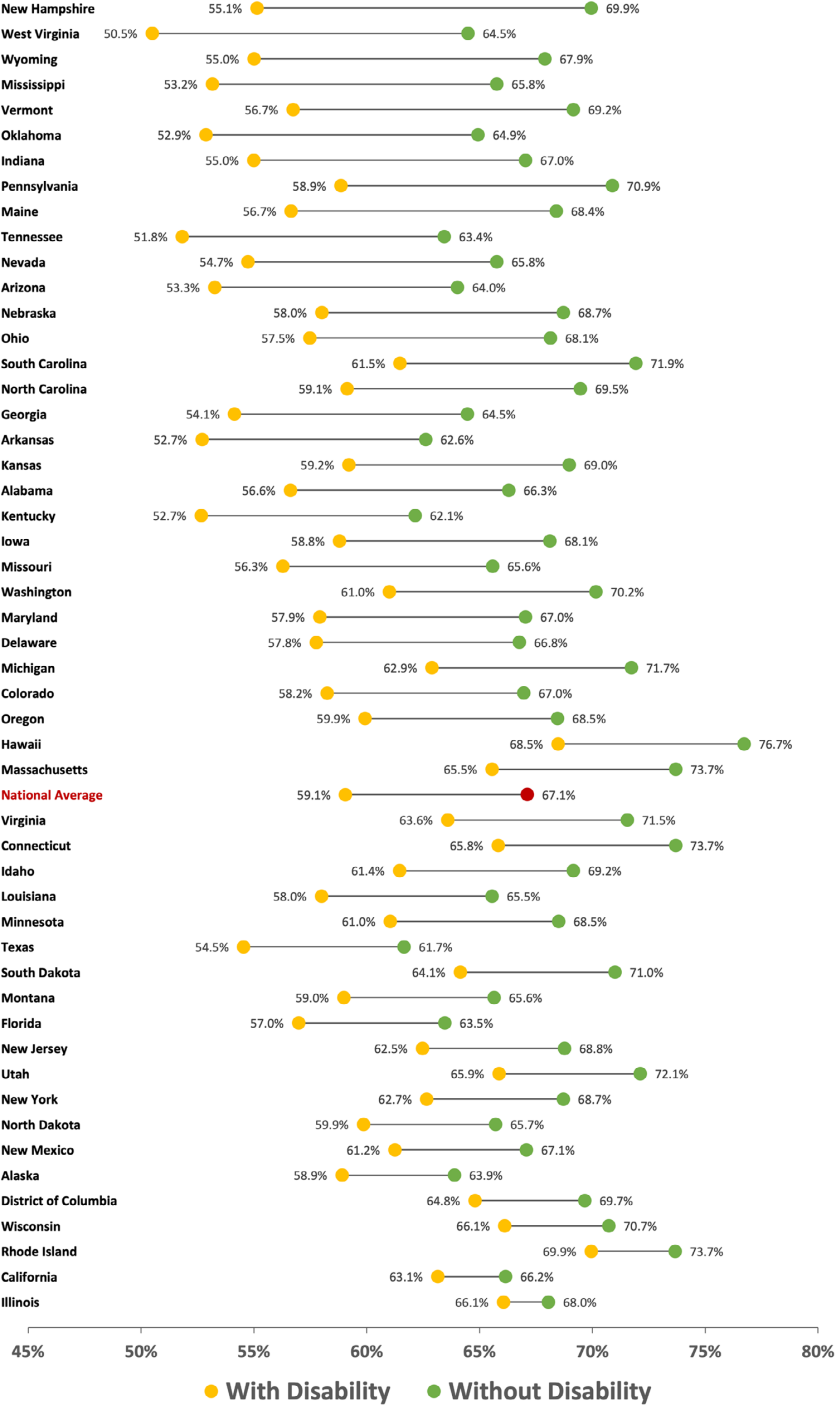
Proportion of dental care utilization within a year, comparing adults with and without disability in each of the 50 states and Washington DC in 2020.

The lowest utilization of dental services within a year among individuals with disabilities was found in the Southern states (Figure 2). After adjusting for sociodemographic factors, the national average of individuals with disability who have had a dental visit within a year was 59.1%, which is 8.0% points less than for adults without a disability (Figure 3). Out of the fifty states and DC, 31 states had greater disparities than the national average between individuals with and without disability, and the gap ranged between 14.8 to 1.9% points. The states with highest dental care utilization within a year for adults with disabilities were Rhode Island (69.9%) followed by Hawaii (68.5%) and Wisconsin (66.1%). On the other hand, the states with the highest utilization among adults without disability were Hawaii (76.7%) followed by Massachusetts and Connecticut, both with (73.7%). West Virginia had the lowest proportion of individuals with disability to have accessed dental care within a year (50.5%), and New Hampshire had the greatest disparity between the two groups (14.8% points). States that showed the lowest disparities between the two groups were Illinois (2.0%), followed by California (3.0%) and Rhode Island (3.8%). The detailed state‐level proportions of time since the last dental visit by disability status are presented in the supplementary document online.

### 3.2 Dentate status

**FIGURE 4:**
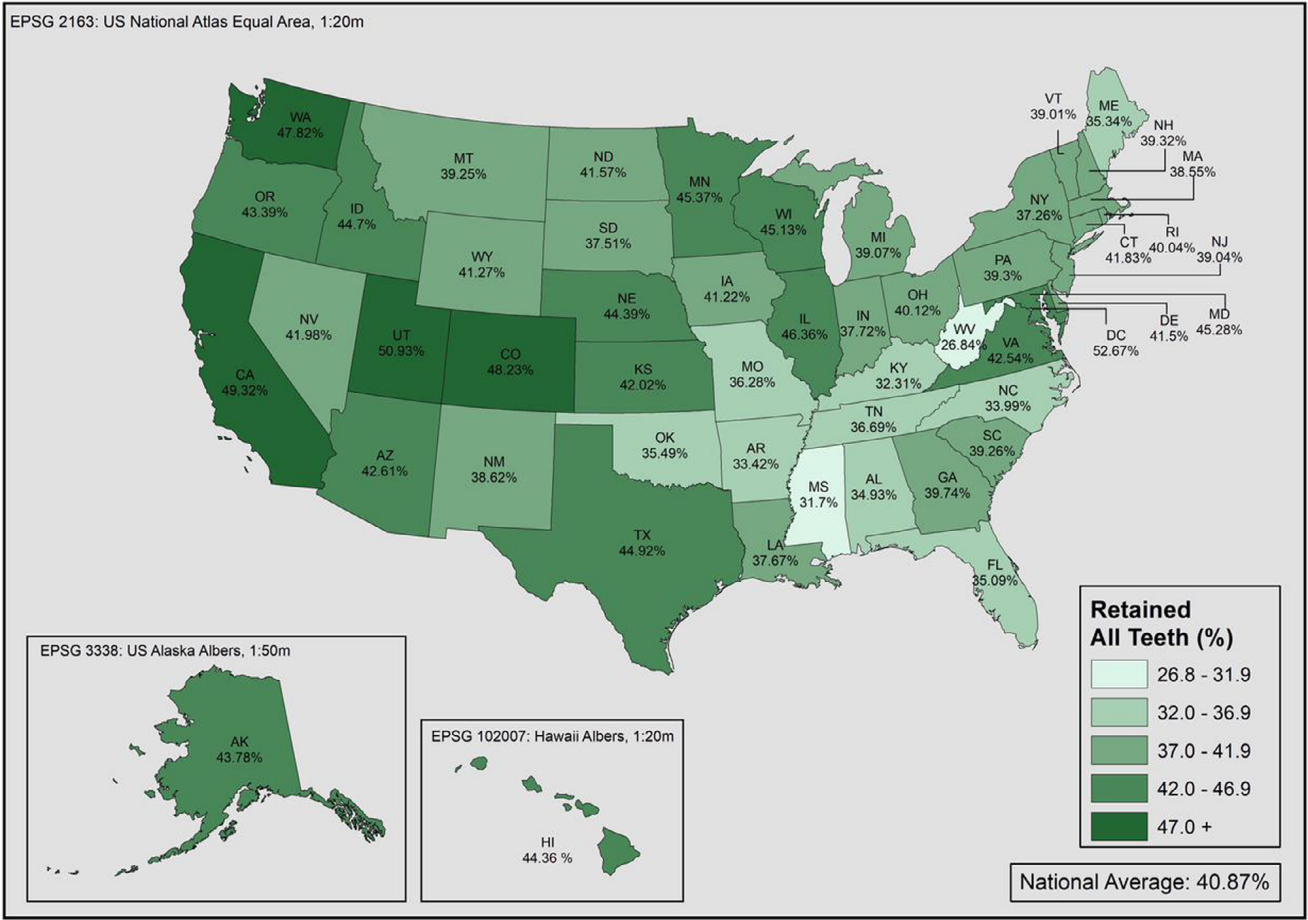
Proportion of adults with disabilities who retained all teeth in the United States in 2020.

**FIGURE 5:**
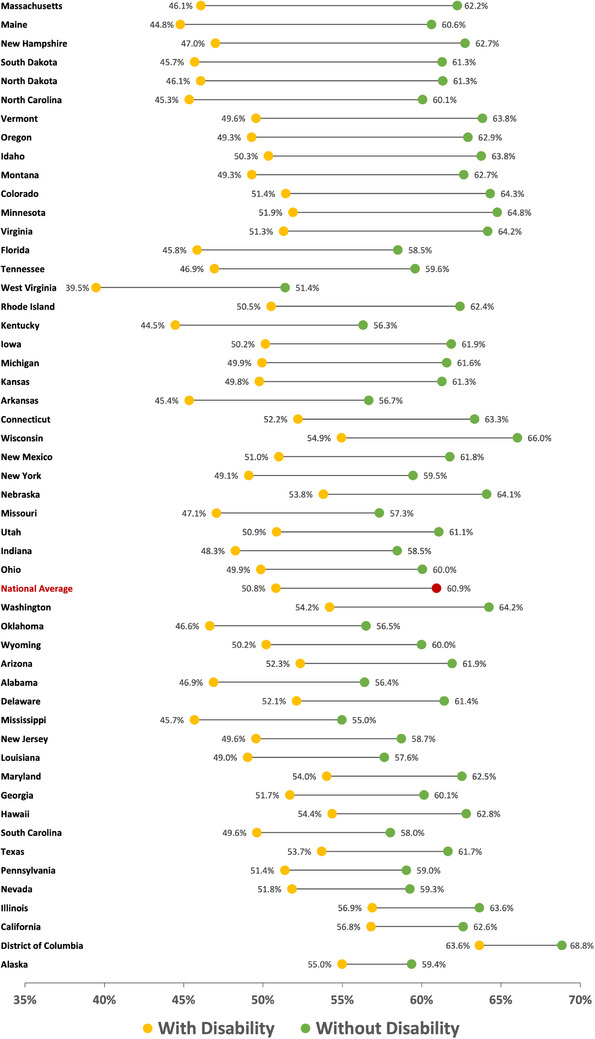
Proportion of retained teeth among adults with disabilities compared to adults without disability in each state and Washington DC in 2020.

The highest proportion of individuals with disabilities who reported complete tooth retention were found in the Western states, while the Southern states had the lowest proportions (Figure 4). Nationally, only 50.8% of individuals with disability had complete tooth retention, which is 10.1% points less than individuals without disability after adjusting for sociodemographic factors (Figure 5). Adults with disability had worse tooth retention in all 50 states and DC compared to adults without disability, with the gap ranging between 16.2 and 4.4% points. Thirty‐one states had greater disparities than the national average. West Virginia reported the lowest proportion of complete teeth retention among both adults with and without disability at 39.5% and 51.4% respectively. Washington DC had the highest proportion of adults with disability who reported having complete dentition (63.6%), followed by Illinois (56.9%) and California (56.8%). In comparison, the highest proportions of complete dentition among adults without disability were also in DC (68.8%), but followed by Wisconsin (66.0%) and Minnesota (64.8%). Massachusetts had the greatest disparity between the two groups (16.2%), while the smallest disparities were found in Alaska (4.4%) and DC (5.2%). The detailed state‐level proportions of missing teeth by disability status are presented in the supplementary document online.

### 3.3 Dental outcomes by type of disability

The most commonly reported disability among US adults was mobility (12.3%), followed by cognition (10.7%), whilst inability to perform self‐care was reported the least (3.3%) (Table 2). In general, adults with a disability were almost two times less likely to have had a dental visit within the past year than those without disabilities. This was true regardless of the type of disability. By the same token, adults with disabilities were twice as likely to have had their last dental visits more than 5 years ago or never compared to adults without disabilities. Furthermore, dental care utilization was highest among adults with a hearing disability (58.4%), whilst individuals with self‐care disabilities were reported to have had the lowest percentage of dental visits within a year (46.2%). Similarly, people without any disabilities were more likely to have retained all their teeth than people with disabilities, regardless of the disability type. Individuals with disabilities related to cognition had the highest percentage of complete teeth retention (45.9%), whilst only 28.4% of those with self‐care disabilities had a complete set of permanent teeth. Similarly, the highest proportion of individuals who reported complete edentulism were among those with self‐care disabilities (16.3%). With increasing number of disabilities in an individual, both the percentage of complete teeth retention and the recent utilization of dental care tend to decrease, reflecting a correlation between increased disabilities and diminished dental health.

**TABLE 2:** Prevalence of disabilities, proportion of dental care utilization, and missing teeth by disability among adults who reported their disability status and completed the oral modules questionnaire in the Behavioral Risk Factor Surveillance System (BRFSS), 2020.

		Time since last dental visit %^a^ (95%CI)			Number of missing teeth %^a^ (95%CI)		
Disability	Prevalence %^a^ (95%CI)	Within a year	1–5 years	5+ years or never	None	Some teeth	All teeth
Hearing							
No	93.5 (93.4, 93.7)	65.4 (65.0, 65.8)	23.9 (23.6, 24.3)	10.7 (10.5, 11.0)	60.3 (59.9, 60.7)	35.4 (35.0, 35.8)	4.2 (4.1, 4.4)
Yes	6.5 (6.3, 6.6)	58.4 (57.0, 59.7)	23.3 (22.2, 24.4)	18.3 (17.3, 19.4)	36.2 (34.9, 37.6)	50.7 (49.4, 52.1)	13.0 (12.3, 13.9)
Vision							
No	95.0 (94.9, 95.2)	65.7 (65.3, 66.1)	23.6 (23.3, 24.0)	10.7 (10.4, 10.9)	59.9 (59.5, 60.3)	35.7 (35.3, 36.1)	4.4 (4.2, 4.5)
Yes	5.0 (4.8, 5.1)	49.5 (47.6, 51.4)	29.3 (27.5, 31.1)	21.2 (19.9, 22.7)	37.0 (35.2, 38.9)	50.0 (48.1, 51.8)	13.0 (11.9, 14,2)
Cognition							
No	89.3 (89.1, 89.6)	66.6 (66.2, 67.0)	23.1 (22.7, 23.5)	10.3 (10.1, 10.6)	60.3 (59.9, 60.7)	35.4 (35.0, 35,8)	4.3 (4.1, 4.4)
Yes	10.7 (10.4, 10.9)	51.5 (50.3, 52.7)	30.1 (29.0, 31.2)	18.4 (17.5, 19.3)	45.9 (44.7, 47.2)	44.8 (43.6, 46.1)	9.2 (8.6, 9.9)
Mobility							
No	87.7 (87.4, 87.9)	66.8 (66.4, 67.2)	23.4 (23.1, 23.8)	9.8 (9.5, 10.0)	62.8 (62.4, 63.3)	33.9 (33.5, 34.3)	3.3 (3.1, 3.4)
Yes	12.3 (12.1, 12.6)	51.5 (50.5, 52.6)	27.1 (26.2, 28.0)	21.4 (20.6, 22.3)	29.2 (28.2, 30.2)	54.9 (53.9, 56.0)	15.9 (15.2, 16.5)
Self‐care							
No	96.7 (96.6, 96.9)	65.5 (65.2, 65.9)	23.7 (23.3, 24.0)	10.8 (10.6, 11.0)	59.7 (59.3, 60.2)	35.8 (35.4, 36.2)	4.4 (4.3, 4.6)
Yes	3.3 (3.1, 3.4)	46.2 (44.2, 48.3)	30.0 (28.1, 31.9)	23.8 (22.2, 25.6)	28.4 (26.6, 30.3)	55.3 (53.3, 57.3)	16.3 (14.9, 17.8)
Independent living							
No	93.4 (93.2, 93.6)	66.2 (65.8, 66.6)	23.4 (23.0, 23.7)	10.5 (10.2, 10.7)	60.3 (59.9, 60.7)	35.5 (35.1, 35.9)	4.2 (4.0, 4.3∖0
Yes	6.6 (6.4, 6.8)	47.5 (46.1, 49.0)	30.7 (29.4, 32.1)	21.8 (20.6, 23.0)	36.9 (35.4, 38.4)	49.1 (47.6, 50.5)	14.0 (13.1, 15.0)
Number of disabilities							
0	74.5 (74.1, 74.8)	68.4 (68.0, 68.9)	22.7 (22.3, 23.2)	8.8 (8.6, 9.1)	64.8 (64.4, 65.2)	32.4 (32.0, 32.9)	2.8 (2.6,2.9)
1	15.0 (14.7, 15.2)	58.6 (57.6, 59.5)	26.0 (25.0, 26.9)	15.5 (14.8, 16.2)	46.5 (45.6, 47.5)	45.4 (44.5, 46.4)	8.0 (7.6, 8.5)
2	5.7 (5.5, 5.8)	50.8 (49.3, 52.2)	29.0 (27.6, 30.4)	20.3 (19.1, 21.5)	37.6 (36.0, 39.2)	32.4 (32.0, 32.9)	2.8 (2.6, 2.9)
3	2.9 (2.7, 3.0)	48.5 (46.2, 50.9)	28.7 (26.7, 30.8)	22.8 (20.9, 24.7)	30.3 (28.0, 25.4)	52.8 (50.5, 55.2)	16.9 (15.3, 18.6)
4+	2.0 (1.9, 2.1)	42.6 (40.1, 45.1)	30.6 (28.4, 33.0)	26.8 (24.7, 29.0)	23.1 (21.0, 25.4)	57.6 (55.1, 60.1)	19.2 (17.5, 21.1)

^a^ Weighted percentages.

## 4 DISCUSSION

### 4.1 Summary and interpretation of the findings

We used the 2020 cycle of BRFSS data to address the gaps in the current literature by investigating the disparities in access to dental care and tooth loss between adults with and without disabilities across various states in the US. In our results, the states with the highest percentage of adults with disabilities were West Virginia, Mississippi, and Kentucky. Colorado, Maryland, and Hawaii had the lowest prevalence. Comparison of states demonstrated a clear gap in the utilization of dental care and in dentate status.

People with disabilities in southern states, including West Virginia, Tennessee, Kentucky, and Mississippi, are less likely to visit the dentist than people without disabilities. This is likely due to a number of factors, including the lack of availability of a sufficient dental workforce in these states. According to a report by the American Dental Association, West Virginia and Mississippi have the lowest dentist to population ratios in the country.^12^ This means that there are fewer dentists per capita in these states, which makes it more difficult for people to find and access dental care.^13^

Furthermore, the prevalence of missing teeth differed in distribution. States with the highest percentage of disabilities (such as West Virginia) had the lowest percent of people with full dentition. Other studies have also showcased this inversely proportional relationship between disability status and teeth retention.^14^ This association can be ascribed to the different obstacles people with disability face, such as communication barriers and the lack of timely access to equipped facilities for more conservative care.^15^

### 4.2 Medicaid

The extent of dental benefits and range of dental services provided by Medicaid may explain the disparity in dental care utilization between adults with and without disabilities among states. For example, Rhode Island's Medicaid program includes a comprehensive adult dental benefit, which covers diagnostic, preventive, restorative, periodontal, surgical, prosthetic, and limited endodontic services.^16^ In contrast, West Virginia's Medicaid program only covers emergent dental procedures for adults 21 years of age and older. This means that adults with disabilities in West Virginia are less likely to have access to preventive and restorative dental care, which can lead to poorer oral health outcomes.^17^ However, it is important to note that coverage alone is not sufficient to ensure access to dental care. Even in states that provide extensive dental benefits via Medicaid, adults with disabilities continue to face barriers to care access. These barriers include a limited number of dentists accepting Medicaid, structural impediments to access, communication challenges, and a lack of perceived need for dental care.

Inadequate training of dentists in managing patients with disabilities may account for a significant portion of the observed disparity in healthcare access, even in states with available dentists accepting generous Medicaid dental benefits for adults.^15^, ^18^ For instance, we found the dental care utilization rate in Massachusetts to be 65.5% among adults with disabilities, a figure notably below the 73.7% rate observed among their peers without disabilities. The Commission on Dental Accreditation published accreditation standards in 2004 that require graduates to be competent in assessing the treatment needs of patients with special needs. Yet, a survey of general dentists in the State of Michigan found that the most common barriers to providing dental care to patients with disabilities were behavior management concerns, inadequate training, and lack of clinical experience.^19^ Further training and better reimbursement were reported in the same study to be the most promising factors to improve the ability of dentists to care for these patients.

### 4.3 Covid‐19

Dental care utilization declined in March 2020 and reached an 80.9% drop by April 2020 compared to the previous year. Southern states like Florida and Missouri had a low level of dental care utilization even after the pandemic, reaching a 12.5% decrease by the end of 2020. These individuals were reported to be more likely to experience surgery visits and less likely to experience preventive and restorative visits.^20^

Although tele dentistry became more common during the coronavirus period, providing dental consultation, instructions, and treatment planning, it coincided with a decrease in the overall number of dental visits. Patients received delayed treatment during this time, which resulted in more invasive procedures. For example, patients with untreated oral abscesses were more likely to have their teeth extracted instead of being treated and retained. Furthermore, as a consequence of continuously postponing dental treatment and apprehensiveness related to the pandemic, the ability to maintain a healthy periodontium decreased, leading to worsening periodontitis and tooth loss. These obstacles during the COVID‐19 period all contributed to the dental status of US individuals.^20^

### 4.4 Strengths and limitations

One of the strengths of using BRFSS data is that it is a nationally representative survey, which means that the results can be generalized to the entire population of the United States. Additionally, BRFSS data is collected using a standardized methodology, which ensures that the data is of high quality and comparable across states and over time. Nonetheless, the cross‐sectional study design does not dictate whether disability occurred before or after our outcomes of interest, which introduces limitations in terms of temporality. In addition, the data collected by BRFSS is reliant on self‐reporting through phone interviews, which may be susceptible to misclassification or recall bias. BRFSS is also limited in its ability to provide information about disparity among children and adolescents. Lastly, the data collected did not provide information on dental insurance, which ultimately prevented us from incorporating this factor in our analysis.

### 4.5 Implications

The results showcase a variation in the distribution of disability across states in the US. The decreased utilization of dental care and the increased number of missing teeth is seen in states with a higher disability prevalence. From these data we can identify which states are most impacted by this disparity and which ones are ahead. Guidance can then be used from such states, for example, Rhode Island, which had the highest proportion of dental care utilization among those with disabilities to improve the implementation of interventions in states like West Virginia, which had the lowest utilization and a large gap between the groups. The importance of our results also lies in leveraging them to target resources and intervention programs to states that need it the most. Improving dental education curricula to include more clinical training, increasing transportation assistance, expanding Medicaid coverage, and raising reimbursement rates for patients with disabilities could lead to increased utilization of dental care and improved oral health in all states. In addition, our study can highlight the states in need of support from large funding organizations to improve their overall dental status. Finally, our data and findings can also be used as a comparative measure to track the effectiveness of interventions and policies in the future.

## 5 CONCLUSION

Similar to findings in the literature, our study demonstrated that adults with disabilities have less access to dental care and experience more oral health problems compared to individuals who don't have a disability.^21^, ^22^ This showcases a need for enhancement of interventions that can improve access to dental care.^23^ However, there is a lack of information in current literature that explores the distribution of disability with respect to access to dental care at a state level. Such data is essential to address limitations in interventions among states. Therefore, our study focused on addressing this need by comparing dental care utilization and dentition status among US adults with and without disability, across states. The findings from our study can potentially contribute to identifying solutions to address the disparity in dental care utilization and dentition status among adults with disabilities at the state level.

## Supporting information



Supplementary information
